# Human Immunodeficiency Virus Infections and Reproductive Health: A French Legal and Ethical Approach

**DOI:** 10.1155/S1064744997000306

**Published:** 1997

**Authors:** Claude Sureau

**Affiliations:** 1 Theramex Institute Bioethics, Women's Health and Society Paris France; 2 147, Avenue de Malakoff Paris 75116 France


Infectious Diseases in Obstetrics and Gynecology 5:199–200 (1997)
© 1997 Wiley-Liss, Inc.

### Human Immunodeficiency Virus Infections and Reproductive Health: A French Legal and Ethical Approach

**Claude Sureau***

*Theramex Institute, Bioethics, Women's Health and Society, Paris, France*

Most of the points raised by Chervenak, McCullough, and Ledger apply to the French position, particularly as far as the historical and psychological aspects are concerned.

We may agree with the possibility and furthermore the need to "rethink the clinical and public health ethics of HIV infection" and to accept "a more ethically balanced approach" than the "exclusive emphasis on the rights of those with HIV infection to the exclusion of their ethical obligations to others."

Every issue dealing with information, persuasion, and possible termination of pregnancy is in accordance with the French approach.

As far as the legal and ethical aspects are concerned, one must point out that France is a smaller country, with a society very centralized in its structure. This is particularly true for legal considerations, the origin of which is to be found in the statute law as expressed in the Civil Code of Napoleon, to ethical considerations regularly expressed by the National Consultative Committee on Ethics in Life and Health Sciences [Comité Consultatif National d'Ethique (CCNE)] founded in 1983, as well as to welfare considerations, through the generalized social security system, which tries to combine (with some difficulty) safety, efficiency, and justice. We shall consider three major issues, mainly the difficult problem when faced with possible conflicts of interest and rights for each member of the couple, and for the unborn child.

The first point concerns the partner. The legal situation is unequivocal: the absolute necessity to keep confidentiality given any medical situation, irrespective of the possible harmful effects to the partner, forces the physician to remain silent. Respect for confidentiality is a strict obligation, established by the civil code, the penal code, and the code for public health. If broken, the risk of prosecution is high. The explanation for this situation has been put forward numerous times, and has to do with the risk of losing the patients' trust if they suspect that confidentiality could be compromised.

Of course, the risk for the partner has been taken into consideration, particularly by the French Academy of Medicine (the highest medical authority, which has the main and official duty to advise the French Government in health matters), which was in favor of an inflection of this rule in very specific circumstances and under very strict control; however, its advice was ignored.

A risk for the physician in charge of both partners could be the possible suing by the noninfected partner, in the case of contamination, for failing to provide advised information. This situation has not yet arisen. However, a practical solution could be to advise practitioners to limit their care to one of the partners in such circumstances.

The second point concerns the child to be. First, let us consider the problem of prenatal testing. The situation is identical to the one prevailing in the United States; i.e., the practitioner has the obligation, both moral and legal, to offer the test to the patient and to try and convince her to accept it. However, this situation is highly paradoxical and questionable, since it is an exception to the prevailing rules concerning prenatal care: for more than 50 years now prenatal care, including some blood tests, has been mandatory in France, for ev-

*Correspondence to: Dr. Claude Sureau, 147, Avenue de Malakoff, 75116 Paris, France.

Received 1 October 1997

Accepted 21 October 1997

ery pregnant woman. If she does not abide by these rules, she is faced with financial disincentives; i.e., she no longer benefits from the "prenatal allowance" offered to every pregnant woman. Among the mandatory tests are, e.g., the tests for rubella, toxoplasmosis, syphilis, hepatitis, the red cell count, the blood groups, etc. It is paradoxical to note that the HIV test is not included and the disease, consequently, is singled out. This situation is a direct result of some "lobbies" fearing that obligatory testing will stigmatize HIV infection. The consequence of their action has resulted in the precise opposite. The French Academy of Medicine once again has firmly expressed its opposition in 1992 and more recently, but has failed to alter matters. This underlines the psychological power alluded to by Chervenak et al. The burden of proof of all medical information is on the shoulder of the practitioner, since a decision (which may still be submitted to change) by the supreme court this year, it follows that the physician must retain a document of proof for 48 years (30 years as usual, plus 18 years till the majority of the child). If treatment is refused, a very rare situation indeed, there is in France no possible recourse for the medical profession other than to try and persuade the patient by all means possible. There is no legal recourse to prosecute for "irresponsible behavior," and no legal recognition of "fetal personality" (the report on the "beginning of life" to the time of fertilization is possible only if the baby is born alive, and if it is beneficial to it, generally for patrimonial reasons). Similarly, to the best of my knowledge, no trial has been initiated by a child against its parents.

A third point may be considered: which attitude to adopt when seropositive parents request medically assisted reproduction. The general attitude prevailing in France is to refuse. However, some changes have been perceived recently. This is particularly the case for the so-called "discordant" couples: if the male partner is positive and the female is negative, attempts have been made, instead of using artificial insemination with donor sperm, to realize a lavage of the partner's sperm followed by insemination, associated with timely ovarian stimulation.

If the female partner is HIV positive and the male is HIV negative, the situation is obviously more difficult. Insemination with the partner's sperm could be carried out after due thought and consideration. The reduced risks for both mother and child to be are valid arguments in favor of this approach; however, it is still a matter of controversy.

Finally, the conclusion reached by Chervenak and others in favor of a more balanced attitude, emphasizing "the beneficence-based and autonomy-based obligations of the physician as well as the beneficence-based obligations of the pregnant patient to the fetal patient" (although the latter is legally deprived of rights), may be ethically shared by the majority of the French medical profession.

